# Gestational Diabetes Mellitus Affects Offspring’s Epigenome. Is There a Way to Reduce the Negative Consequences?

**DOI:** 10.3390/nu12092792

**Published:** 2020-09-11

**Authors:** Monika Słupecka-Ziemilska, Piotr Wychowański, Monika Puzianowska-Kuznicka

**Affiliations:** 1 Department of Human Epigenetics, Mossakowski Medical Research Centre, Polish Academy of Sciences, 5 Pawinskiego Street, 02-106 Warsaw, Poland; mpuzianowska@imdik.pan.pl; 2 Department of Oral Surgery, Medical University of Warsaw, Binickiego 6, 02-097 Warsaw, Poland; piotrwychowanski@wychowanski.pl; 3 Department of Geriatrics and Gerontology, Medical Centre of Postgraduate Education, 61/63 Kleczewska Street, 01-826 Warsaw, Poland

**Keywords:** GDM, diabetes, programming, epigenetic programming

## Abstract

Gestational diabetes mellitus (GDM) is the most common pregnancy complication worldwide and may result in short-term and long-term consequences for offspring. The present review highlights evidence of epigenetic programming, mostly from human studies, which occurs in offspring exposed to maternal GDM during different stages of development, paying special attention to the differences in sensitivity of offspring to maternal hyperglycemia as a result of sex-related factors. We also aim to answer the following question: If these epigenetic changes are constant throughout the lifetime of the offspring, how do they present phenotypically?

## 1. Introduction

**Figure 1:**
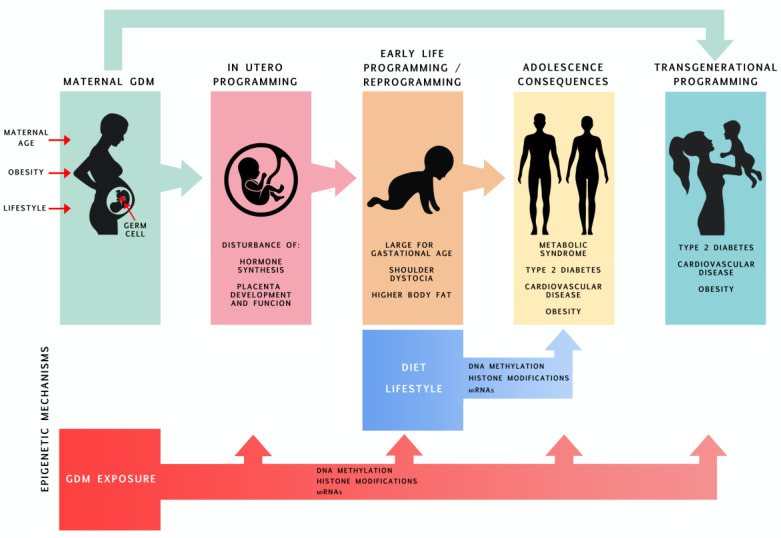
Consequences of offspring being exposed to maternal GDM throughout its lifetime with an emphasis on epigenetic programming.

The first 1000 days, which starts from pre-conception until approximately two years of age, is the period during which tissues and organs are sensitive to several environmental factors which can shape their development and maturation and consequently may program their susceptibility to diseases later in life. This phenomenon has been termed the Developmental Origins of Health and Disease theory [1]. Continuous efforts are being made to gain knowledge concerning the mechanisms by which tissues and organs may be controlled during this fragile period of development. Maternal obesity, gestational diabetes mellitus (GDM) and other chronic diseases, as well as the physical and mental condition and nutrition of the mother during pregnancy, along with environmental factors such as pollution, smoke or medications, all affect phenotypic outcomes through modification of the epigenome of the developing organism, a process called “epigenetic programing”. In this review, we first briefly present data regarding GDM epidemiology and its consequences for offspring, and we describe the epigenetic modifications and mechanisms of their action (Figure 1). Next, we present a detailed overview of the current state of knowledge on epigenetic changes in offspring exposed to maternal GDM and how these epigenetic changes possibly relate to increased susceptibility of offspring to chronic diseases in adulthood.

## 2. Gestational Diabetes Mellitus—A Brief Description of GDM Epidemiology and Consequences of GDM for Offspring

Gestational diabetes mellitus (GDM) is defined as any degree of glucose intolerance that was first recognized during pregnancy, regardless of the degree of hyperglycemia. However, it should be highlighted that, according to the very recent recommendations of the American Diabetes Association (Standards of Medical Care in Diabetes, 2020) [2], this definition has some limitations and further work is required in this area. The main concern relates to the correctness of the diagnostic criteria that are currently used. GDM affects between 1.8% and 31.5% of pregnancies worldwide, depending on the heterogeneity of screening approaches, diagnostic criteria and population characteristics, and its prevalence has increased constantly over the past few decades [3]. The increasing number of mothers affected by GDM is probably related to advanced maternal age, which, in turn, is associated with the increasing risk of obesity and overweight, which makes mothers more susceptible to hyperglycemia during pregnancy. A very recent retrospective cohort study of Malaysian women (SECOST) showed that overweight or obesity and age ≥ 35 years were associated with a 2.45-fold higher risk of GDM [3]. Excessive gestational weight gain during the second trimester by these women further increased their risk of developing GDM [4]. Moreover, other factors such as unhealthy nutritional habits (so-called “western diet” composed, to a large extent, of highly processed and calorie-rich foods, with low amounts of vegetables) and low physical activity are also significant risk factors for developing GDM [5,6]. Maternal hyperglycemia affects the fetus’s hormonal response and its insulin secretion, which is crucial for fetal organogenesis and growth. As a result, GDM increases the incidence of short-term and long-term complications observed in the offspring. The short-term consequences of GDM include large-for-gestational-age infants, increased prenatal and perinatal mortality, prematurity, higher risk of cesarean section and increased perinatal injuries (i.e., shoulder dystocia). The long-term effects of GDM in offspring include a higher risk of developing obesity, metabolic syndrome, diabetes mellitus type 2 (DM2) and cardiovascular disease later in life [7]. There is also growing evidence linking GDM with abnormal brain development, with consequences including problems with attention [8] and general cognition [9].

### Gender and Sensitivity to GDM Exposure

Consequences of GDM are dependent on the sex of the offspring. This phenomenon possibly originates from the number of transcripts located on sex chromosomes, which in turn regulate autosomal gene expression [10]. A finite set of genes on human X chromosomes escape inactivation and would therefore be expected to exhibit higher expression levels in XX compared to XY cells. According to studies on mice models [11,12], the number of genes on the X chromosome that escape inactivation affect adiposity and food intake, suggesting that genes expressed on the X chromosome contribute to sex differences in obesity and metabolism. Moreover, many studies suggest that the placenta is the major source of sex differences in metabolic phenotypes as its development starts very early during fetus development from extra-embryonic tissues but, genetically, it “has the same sex” as the fetus. Furthermore, sex-dependent development and growth of the placenta has been reported, where male placentas tend to be smaller, which makes male fetuses more vulnerable in the face of environmental changes [13].

There are also sex differences in circulating hormone levels and the sensitivity to hormones observed both during prenatal and postnatal development in animals. Metabolic hormones and adipokines (e.g., leptin, ghrelin, insulin), which also play integral roles in the development of major hypothalamic pathways required for maintaining energy homeostasis and in responses to changing nutrient status (e.g., glucose levels), are possibly responsible for some of the phenotypic sex differences observed in response to changes in nutrition during early life [14].

Disturbances in the synthesis of sex hormones in response to a maternal high fat diet have previously been reported in animal studies [15]. Fetal sex hormones are involved in the sex-specific metabolic disturbances, as they are responsible for maintaining energy metabolism and body composition. In adult life, as recently discussed by Mauvais-Jarvis [16], testosterone deficiency contributes to the development of hyperglycemia and diabetes in men, while estrogen deficiency was reported to increase diabetes risk in women due to alterations in insulin secretion, insulin sensitivity and glucose effectiveness.

More studies are needed to fully understand how GDM can differentially impact the long-term health of male and female individuals. According to a very recent review by Nijs et al., among 23 studies published between the years 2000 and 2019, which aimed at evaluating the long-term metabolic risk in offspring from mothers suffering from GDM, only seven evaluated possible sex differences [17]. However, most of the available human studies have shown that female children are more susceptible to metabolic programming as a result of a diabetic mother than male children [18,19,20,21]. Females may also be more sensitive to maternal glucose intolerance [19]. Moreover, the Danish National Birth Cohort study revealed that GDM is associated with earlier onset of puberty in girls [22]. However, data from the OBEGEST cohort study show that GDM is a risk factor for childhood overweight in boys but not in girls [23].

## 3. Epigenetic Mechanisms of Programming

Epigenetics is the study of mitotically and meiotically heritable changes in gene function that do not involve changes in the DNA coding sequences. The classical epigenetic mechanisms include DNA methylation and histone modifications, but noncoding RNA including micro-RNAs (miRNAs) have been also classified by most authors as epigenetic modulators as they affect the protein levels of target genes without modifying their DNA sequence. DNA methylation is the most extensively studied epigenetic mechanism. During DNA methylation, the covalent transfer of a methyl group occurs into the 5′ position of cytosine residues. Methylated cytosines are found primarily at the cytosine-guanine dinucleotides (CpG) but are also observed at non-CpG sites (CpA, CpT, and CpC), both being important epigenetic determinants of gene expression and genomic stability [24]. In general, hypermethylation of DNA when located in a gene promoter is associated with gene silencing, while a low level of methylation coincides with gene activation. Changes in the methylome are crucial for mammalian development and cell differentiation. Before the blastocyst forms, genome-wide DNA methylation drastically changes from a hypermethylated state to almost 0% methylation. Thereafter, DNA methylation increases in a tissue-specific manner during the remainder of the pregnancy [25]. As development progresses, the increase in DNA methylation preferentially occurs near genes involved in general developmental processes, whereas loss of DNA methylation is mapped to genes with tissue-specific functions. However, dynamic DNA methylation is associated with all regions of the gene and there are many ways by which this modification can regulate gene expression during and after development. There is evidence that methylation can be both positively and negatively associated with the expression of genes. Moreover, intragenic DNA methylation has been implicated in assisting with the choice of exon–intron boundaries during co-transcriptional splicing of pre-mRNAs. However, there is also evidence which suggests that many changes in DNA methylation during development do not correlate with changes in expression of the associated gene [26].

Although the majority of studies on epigenetic programming have focused on DNA methylation, there are also data focusing on the role of histones with regard to developmental programming. Histones, which condense DNA and package it into the chromatin, may undergo several types of modification, e.g., methylation, acetylation and phosphorylation, which influence chromatin structure and affect gene expression. According to the current state of knowledge, histone modifications are key structural alterations that can either promote or repress gene expression, depending on the location (lysine or arginine residues in the histone), whereas histone acetylation is mostly associated with the active euchromatin state [27]. Differently from DNA methylation and histone modifications, which modulate gene transcription, microRNAs are a class of small, noncoding RNAs that regulate gene expression at the post-transcriptional level. Each microRNA affects several target messenger RNAs (mRNAs) through their binding to the 3′UTR of the mRNA within the miRNA seed region [28]. A single miRNA can directly downregulate the production of hundreds of proteins [29]. Moreover, miRNAs and their target genes can be mutually regulated.

## 4. The Effect of Exposure to GDM on the Epigenome of Offspring throughout Their Lifetime

In order to minimize selection bias in assessing the current state of knowledge regarding the effects of GDM exposure on epigenetic changes in offspring, we searched PubMed using “gestational diabetes mellitus AND epigenetic” as keywords. Out of 314 articles, of which 131 were reviews, we selected 40 original articles written in English which were related to epigenetic changes in the offspring of GDM mothers. We then excluded two studies involving mothers with pre-existing diabetes and another two studies due to a small number of subjects. Out of the remaining 36 articles, 26 of them described studies related to our topic and were conducted on humans and they were all included in the current review. The remaining 12 articles described data obtained from animal models and five of them were included for completeness of human data or to supplement data regarding transgenerational programming.

### 4.1. Fetal Epigenetic Changes Following Exposure to GDM

During fetal development, the placenta plays a key role, not only in controlling the process of exchange between mother and fetus, thus enabling fetal growth and development, but also through active participation in maternal metabolism and contribution to fetal programming. The anatomy and physiology of the placenta ensures appropriate nutrient supply to the fetus, thus maintaining fetal growth and development. Epigenetic regulation has been shown to be crucial for both placental development and function (for overview, see [30]). For example, a recent study on the role of circulating miRNAs encapsulated in extracellular vesicles (EVs) identified a group of ten maternal miRNAs which are involved in trophoblast proliferation/differentiation and are elevated in GDM mothers. Bioinformatics analysis revealed that these miRNAs are also involved in the regulation of insulin secretion and transport of glucose during pregnancy [31]. It has also been demonstrated that, in response to GDM, the placenta adapts to the availability of nutrients through its DNA methylation [32,33,34]. A study on over one thousand human placental samples revealed that maternal GDM is associated with global DNA hypermethylation [35]. Other studies demonstrated changes in the methylation of several placental genes, including those for regulation of newborn brown adipose tissue and beige adipocytes (leptin (*LEP)*, adiponectin (*ADIPOQ*), peroxisome proliferator-activated receptor gamma coactivator 1-alpha (PGC-1α) encoded by *PPARGC1A)* [36,37,38,39], responsible for the transport of cholesterol and HDL formation (ATP-binding cassette transporter A1 (*ABCA1*) [40], transport of lipids (lipoprotein lipase (*LPL*)) [41]), and key inflammatory genes [42]. Altogether, these findings suggest a strong association between GDM exposure and the regulation of genes related to metabolic disorders [43]. Moreover, the downregulation of histone H3 lysine 9 acetylation (H3K9ac) was reported in GDM placentas, confirming that histone modification can be an important factor in fetal programming in GDM [44].

There are also studies showing other epigenetic changes during development under hyperglycemic conditions, using cell lines of embryonic and fetal origin. Studies on human embryonic stem cells, used as a model for studying early pancreas development, revealed that hyperglycemic conditions impair the formation of definitive endoderm, which is an early intermediate stage of the pancreatic lineage. This process occurs via the modification of histone H3 methylation, suggesting a critical role of chromatin modifications in the developmental process [45].

Moreover, miRNAs can also serve as intermediates between GDM and abnormal development. Fetal epigenetic changes in endothelial cells are suggested to be responsible for the increased risk of cardiovascular disorders and incidence of T2DM later in life [46]. Studies on human fetal endothelial cells from the umbilical cord vein, isolated from GDM pregnancies and healthy pregnancies exposed to high d-glucose concentrations for 48 h, demonstrated an increase in miR-101 expression and a decrease in expression of the enhancer of zester homolog 2 (EZH2), which is involved in the complex initiation and maintenance of the methylation of histone H3 on lysine 27, which in turn results in miR-101 downregulation. These results suggest the existence of a homeostatic regulatory feedback loop between miRNAs and histone methylation. Interestingly, this study also demonstrated decreased survival and functional capacity of cells isolated from GDM mothers in comparison to those isolated from healthy and nondiabetic mothers, even after exposing the cells to normal glucose concentrations for the next 5–6 passages, suggesting the existence of metabolic memory [47].

Another study on human primary feto-placental endothelial cells focused on differences in miRNA expression in relation to the sex of the fetus. Using next-generation miRNA sequencing, the authors showed that the effect of maternal GDM on miRNA signatures was highly dependent on fetal sex. Separate analysis revealed that female fetuses were more susceptible to the mother’s GDM condition than male fetuses, as they responded to the hyperglycemia with changes in the expression of 22 miRNAs, while male fetuses only exhibited changes in the expression of four miRNAs. The biological functions regulated by these miRNAs are related to the well-known adverse functional consequences of diabetes on the endothelium [48].

Interesting observations were made using a mouse model of streptozotocin-induced (STZ) diabetes. Yu at. al. [49] demonstrated that GDM enhances embryonic and neural stem cells’ histone 3 acetylation by the inhibition of histone deacetylases SIRT2 and SIRT6, resulting in neural tube defects in the offspring.

### 4.2. Screening for GDM-Induced Epigenetic Modifications at Birth

The majority of studies on developmental programming are undertaken on umbilical cord blood. Cord blood offers a unique screening opportunity since it is established in utero and may mediate associations of maternal conditions and exposures. Previous DNA methylation studies on cord blood found an association between GDM-induced changes in methylation and early childhood weight [50] and adiposity [51] or brain-related conditions including impairments in neurodevelopment [52] and child depression [53]. A large epigenome-wide DNA methylation study from the Pregnancy and Childhood Epigenetics (PACE) consortium was analyzed for an association between maternal GDM and cord blood methylation and showed that GDM is associated with lower methylation within two genes: the promoter of *OR2L13*, a gene associated with autism, and the gene body of *CYP2E1*, which encodes WHAT and is upregulated in type 1 and type 2 diabetes [54]. Similar findings were published by Weng et al., who showed that exposure to GDM results in changes in the methylation of cord blood cell genes which are strongly linked to type 1 diabetes mellitus, as well as neuronal development-related pathways associated with depression, attention deficit hyperactivity disorder, autism and intellectual impairment [55].

### 4.3. Studies on Adolescent Offspring

A genome-wide DNA methylation study on peripheral blood in adolescent offspring (9 to 16 years old) of women with GDM within the Danish National Birth Cohort showed that in utero exposure to hyperglycemia is associated with DNA methylation abnormalities resulting in 76 differentially methylated CpG sites. In total, 92% of these CpGs showed lower DNA methylation in the GDM offspring than in controls [56]. Moreover, this study also showed that the majority of differentially methylated CpGs among the GDM offspring appear to be confounded by the co-existence of maternal obesity and thus they should also be associated with pre-pregnancy maternal BMI rather than GDM alone. Exposure to GDM and maternal obesity impacts multiple gene-associated networks, including lipid metabolism, developmental disorders, endocrine disorders, regulation of embryogenesis, cardiogenesis and vascular development. All these pathways are also possible mechanisms which could be associated with the development of diabetes and obesity (diabesity). These findings suggest that differentially methylated CpGs might offer some insight into the development of patho-physiological processes. This study also showed that maternal GDM and obesity are more associated with the offspring’s DNA methylation profile than the offspring’s own BMI at adolescence. The results from the Cambridge Baby Growth Study also suggest the existence of postnatal epigenetic stability, since the two genes (*PRKCZ* and *PRKAR1B*) observed to be methylated in GDM offspring during adolescence were also previously shown to be methylated at birth in the cord blood of GDM offspring [57].

The abovementioned study was performed on peripheral blood samples, a model which possesses some limitations since the peripheral blood contains of a mix of different cell types. The authors admitted that a small difference in methylation in the blood (1% to 5%) might reflect a larger difference in a more metabolically relevant tissue (i.e., pancreas).

Other valuable results came from a study conducted on peripheral blood leukocytes collected from siblings [58]. According to previous results [59], the methylation pattern of peripheral blood leukocytes was proven to represent DNA methylation of metabolically relevant tissues, such as the liver, adipose tissue and pancreas. Moreover, studies on sibling pairs with different degrees of exposure to maternal GDM enable us to elucidate epigenetic markers which are specific to intrauterine hyperglycemia, minimizing the confounding effects of genetic variation observed in studies on unrelated offspring. A study on peripheral blood leukocytes from GDM and non-GDM offspring revealed the presence of 12 CpG sites associated with exposure to GDM. Among the differentially methylated CpG sites, epigenetic markers were found at human hepatocyte nuclear factors 4α (*HNF4A)* and at the ras responsive element binding protein 1 (*RREB1)*, associated with diabetes and obesity. Interestingly, differentially methylated CpG sites were also found within catenin delta 2 (*CTNND2)*, which encodes for δ-catenin and is involved in brain and eye development but also overexpressed in cancers [58].

### 4.4. Studies on Adult Offspring

The research discussed above regarding epigenetic changes after exposure to GDM during fetal development (studies on placenta), at birth (cord blood studies) or in the offspring during childhood raises the question of whether these changes are permanent across a whole lifetime and how important they are for the development of age-related and other diseases. The most well-known human studies which seem to confirm the existence of such associations are those regarding adults whose mothers experienced starvation while pregnant during WW II (the Dutch Hunger Winter). These studies showed that exposure to starvation in utero increases the risk of various adverse metabolic and mental phenotypes, such as coronary heart disease, type 2 diabetes, schizophrenia and depression [60]. Moreover, the data collected indicate widespread and persistent changes in DNA methylation, which are also dependent on the sex of the in utero-exposed individual and the timing of exposure during gestation [61,62]. Other data regarding the consequences of maternal hyperglycemia during pregnancy are available from observations on Danish pregnant women during the years 1978–1985 [63]. Biopsies of skeletal muscles were collected from their offspring (aged 26–35) and analyzed for the expression of miR-15 and miR-15b, which were previously shown to be related to the insulin signaling pathway and involved in the development of insulin resistance. This study showed a significant increase in the expression of the miRNAs studied in adult offspring exposed to maternal GDM. Moreover, in another study on the same population of offspring exposed to GDM, a significant increase in DNA methylation within the thioredoxin-interacting protein (*TXNIP*) gene was shown, together with its decreased expression in subcutaneous adipose tissue samples. Increased *TXNIP* expression was shown to inhibit insulin-mediated glucose uptake in skeletal muscle and adipocytes. However, in this study, no changes were observed in the muscles [64]. It should also be mentioned that valuable data can be extracted from the Danish National Birth Cohort (dnbc.dk). The platform was established to investigate the consequences of early life exposure for the risk of developing disease later in life but can also be useful for studies on disease prevention. This project enrolled a large cohort of pregnant women, with long-term follow-up of the offspring. Data collection in this project started in 1996, so the oldest offspring are over 20 years old now. The data collected, as well as the bank containing the biological specimens, are open for access by researchers.

#### Animal Models as Alternatives for Studies on Adult Offspring

It should be emphasized that studies on adult offspring in the context of epigenetic programming poses several limitations due to the huge number of environmental factors affecting the epigenome during life. Full control of environmental conditions, as well as the possibility of planning invasive endpoints, with unlimited tissue sampling, favors studies on animal models. The majority of human studies on the effects of maternal GDM on the development of diabetes in the offspring are performed using placenta, umbilical cord blood or maternal peripheral blood. Although these types of samples enable noninvasive sampling, they may not be a true reflection of the epigenetic changes which occur in more metabolic tissues, due the fact that DNA methylation profiles vary in different tissues. Therefore, samples from the pancreas, for example, which is directly related to diabetes, serve as a good model for studying the role of DNA methylation or other epigenetic modifications in the transmission of metabolic properties from mother to child. It is worth mentioning here the comprehensive study on mice adult offspring, exposed to intrauterine hyperglycemia, which revealed an altered DNA methylation profile in the pancreas of the offspring. Among the genes with differentially methylated regions were those involved in glycolipid metabolism and the related signaling pathways. Some of them were previously reported in human studies, for example, *Hnf1b*, belonging to the family of transcription factors *Hnf1* (human hepatocyte nuclear factors1), which are involved in the development of the liver, kidneys and intestine, as well as in glucose transport and metabolism [65].

### 4.5. Do the Epigenetic Changes Acquired Due to Maternal GDM Exposure Have Long-Lasting Effects?

Some authors suggest that the epigenetic modifications (and their transcriptional results), once programmed, can still be diluted and possibly erased over time under particular circumstances, such as with the use of drugs or lifestyle changes. Resolution of the consequences of in utero exposure to GDM has been observed in studies on animal models, where precise control of the environment, including the diet, allow unbiased conclusions. In a mouse model of GDM, maternal GDM had no long-lasting effects on offspring weight as long as they were maintained on a control diet; however, when they were fed a high fat and high sugar diet (HFHS), the weight of offspring from GDM dams was significantly elevated in comparison to control animals. Interestingly, although increased adiposity was observed in GDM-exposed offspring of both sexes at 4, 12 and 20 weeks of age, adult offspring (31 weeks) exhibited increased adiposity only if they were fed with a HFHS [66]. These data are in agreement with numerous previous studies suggesting that critical phases in the process of developmental programming are conditions influencing both prenatal and postnatal life. Nutrition, specifically maternal breast milk, plays a crucial role in metabolic programming during the early postnatal period.

Two meta-analyses showed that breastfeeding reduces the risk of developing childhood obesity and suggested that the longer the period of breastfeeding, the lower the risk of becoming overweight [67,68]. Similar conclusions came from studies on the GDM population. Observations performed on participants in the Nurses’ Health Study II showed that children exposed to prenatal maternal diabetes or GDM, who were breastfed during the first 6 months of life, had lower risk of being overweight as compared to those fed with formula [69]. Similar observations were made in a group of Hispanic, low-income youth exposed to maternal GDM. Between 2 and 4 years of age, children who were breastfed for 12 or more months had a 72% lower prevalence of obesity than neonates fed with formula [70]. The well understood health benefits of breastfeeding are due to the unique composition of mother’s milk which, apart from the content of macronutrients (i.e., oligosaccharides), is perfectly matched to the growing infant’s needs, since it contains growth factors and hormones involved in the regulation of appetite and energy expenditure. Several previous studies either documented differences or did not notice any significant changes in the composition of the milk from healthy mothers compared to that from those suffering with GDM. Thus, whether these possible differences in human milk composition affect child health and development remains controversial [71].

The role of mother’s milk in epigenetic programming has gained attention due to observations that milk contains the greatest amount of RNAs and miRNAs among body fluids [72]. Moreover, most of the miRNAs in milk are successfully taken up by the infant’s cells, as they are transported in exosomes and the lipid bilayer protects them from digestion and degradation in the infant’s gastrointestinal tract [73]. Therefore, the exosomes allow for postnatal communication between mother and child and may also transfer important regulatory signals necessary for proper growth and development. In contrast, milk formulas are highly deficient in these molecules [74]. A well-studied example of epigenetic regulation in the infant, via milk miRNAs, involves the family of miRNA-148 and -152. These highly conserved molecules were shown to inhibit the activity of methylotransferase 1 (DNMT1), leading to the overexpression of forkhead box P3 (FOXP3), a transcription factor, which in turn results in overexpression of genes related to development (*INS*, *IGF1*, *FTO* and *NRF2*) [74,75]. Is it possible that the beneficial effects of breastfeeding may contribute to breaking the cycle of diabetes between the diabetic mother and child? Further studies are needed which are focused on the miRNA content in the milk from GDM mothers in order to better understand the effects of breastfeeding on the growth of children born to mothers with GDM.

Donovan and Cundy [76] reviewed the available evidence from animal studies, human observational studies, systematic reviews and experimental trials that addressed the impact of diabetes on the future risk of obesity and/or glucose intolerance in the offspring. The authors suggested that the majority of studies analyzing the prevalence of obesity and diabetes in the offspring of GDM mothers did not take into account maternal and paternal BMI. The few studies that did take it into account observed that the differences between GDM-exposed and control offspring diminished, suggesting an effect of postnatal socioeconomic circumstances (including diet and lifestyle) rather than the genetic or epigenetic inheritance of diabetes and obesity.

There are data showing a family-specific histone methylation pattern [77]. Results from male relatives from seven different families indicated peaks in H3K27 and H3K4me3, which were enriched on the genes associated with metabolic glycogen pathways and lipid metabolic pathways. These results suggest either that members of the same family share lifestyle and dietary habits which significantly and similarly influenced their epigenomes or that the results constitute evidence of intergenerational inheritance. Possibly, both mechanisms contribute to the occurrence of similar epigenetic changes. For the change, the existence of transgenerational inheritance was supported by several animal studies, allowing comprehensive analysis through generations. According to this concept, phenotype changes observed in the F2 generation during their lifetime are caused by conditions affecting the F0 generation during pregnancy. At the root of this concept are epigenetic modifications (DNA methylation and noncoding RNA regulation) which occur during the development of germ cells and preimplantation, where the embryo of the F1 generation is exposed to the adverse maternal (F0) intrauterine environment [78]. Although, for the purposes of this review, we focused on maternal, intrauterine conditions, the existence of paternal inheritance has also been shown to play an important role in the epigenetic modifications which affect male germ cells [79]. Ding et al. [80] showed that maternal hyperglycemia (induced by STZ) in a mouse model during pregnancy (F0) did not affect the birth weight of F1 but increased the birth weight of F2 offspring born to F1 offspring exposed to maternal hyperglycemia. The exposure to maternal hyperglycemia also triggered the transgenerational transmission of glucose intolerance and abnormal insulin levels, with evidence of greater susceptibility to development of these conditions in F2 males. The authors reported on the epigenetic modifications responsible for the observed changes, including hypermethylation within the *Igf2* (insulin-like growth factor 2) and *H19* genes, that significantly affected their expression.

Epigenetic markers persisting for generations supposedly enable subsequent generations to better adapt to changing environmental conditions, but sometimes, as a result of adverse environmental conditions during pregnancy, this could lead to maladaptive consequences for the offspring phenotype. However, there is no consensus as to whether transgenerational epigenetic inheritance is also true for humans, who are likely to live in different environments. Horstkemke [81] suggested that the transmission of epigenetic information between generations reduces developmental plasticity and channels the development of offspring in, a particular direction; so, if the “anticipated” environment does not match the actual environment, the offspring will be less adapted and have reduced reproductive success. Therefore, he speculates that the transgenerational transmission of culture (by communication, imitation, teaching and learning) surpasses the effects of epigenetic inheritance and plays an important adaptive role in the evolution of the human species. In our opinion, the role of conscious choice plays an important role in the process of the modification of the human epigenome, including choice of partner, choice of environment and lifestyle, as well the decisions to undergo medical intervention, use drugs or change the current lifestyle.

## 5. Conclusions

Growing evidence shows that, although GDM develops relatively late in pregnancy, it significantly impacts the offspring epigenome, which, in turn, may have health consequences. Therefore, careful diagnosis of hyperglycemia during pregnancy is crucial and all efforts should be directed toward studies on effective and safe interventions which regulate maternal insulin secretion. Although questions regarding the critical windows of development during which strategies and interventions should be implemented to prevent unfavorable epigenetic programing, and the sustainability of epigenome changes later in life and over the next generations are still unanswered, the potentially reversible character of epigenetic modifications encourages further studies. Moreover, as previously suggested [82], epigenetic markers appearing in the epigenome of the GDM offspring can become an effective tool in the early detection and prognosis of adverse phenotypic outcomes.
